# A seven-year analysis of the role and impact of a free community eye clinic

**DOI:** 10.1186/s12909-021-03026-7

**Published:** 2021-12-02

**Authors:** Lucas W. Rowe, Melanie Scheive, Hanna L. Tso, Patrick Wurster, Nicholas E. Kalafatis, David A. Camp, Avrey Thau, Chi Wah Rudy Yung

**Affiliations:** 1 Department of Ophthalmology, Indiana University School of Medicine, Indianapolis, IN USA; 2 Department of Ophthalmology, Baylor College of Medicine, Houston, TX USA

**Keywords:** Free clinic, Urban health, Vision screening, Vulnerable populations

## Abstract

**Background:**

The Indiana University Student Outreach Clinic (IUSOC) Eye Clinic is a monthly student-run eye clinic that provides free visual screening to the Near East Side community of Indianapolis, IN, USA. Screening includes assessments of visual acuity, intraocular pressure, peripheral visual fields, refraction, and non-mydriatic fundus photography.

**Methods:**

This is a retrospective chart review of 875 patients seen at the IUSOC Eye Clinic from October 2013 to February 2020. Data on demographics, insurance coverage, ocular history, physical examination, suspected diagnosis, referral status, and glasses provided were collected and analyzed.

**Results:**

875 patients were seen at the IUSOC Eye Clinic from October 2013 to February 2020. 39.2% of the patients seen at the clinic reported being uninsured. 61.4% of patients were found to have visual acuity of 20/40 or worse, while 51.3% of patients were found to have a near visual acuity of 20/40 or worse. 20.3% of patients were referred to the local county hospital for further evaluation by an ophthalmologist, 14.4% of patients received free glasses prescriptions, and 27.9% of patients received free reading glasses. Common reasons for referral for further ophthalmology evaluation included glaucoma, decreased visual acuity, and diabetic retinopathy. An estimated value of services provided over the seven years of the clinic was 1271 relative value units.

**Conclusion:**

The IUSOC Eye Clinic fills an important role in advancing ocular health and preventing irreversible blindness in an underserved Indianapolis community. Additionally, the clinic demonstrates an educational model for involving medical student volunteers.

## Background

Access and affordability of healthcare has improved and resulted in a reduction in socioeconomic disparities in recent years in the United States because of the Affordable Care Act [1]. In Indiana specifically, this legislation has led to the expansion of Medicaid eligibility through the Healthy Indiana Plan 2.0 to increase healthcare coverage statewide [2]. However, there remain persistent gaps in access to healthcare, including specialized ophthalmic services. Among those with Medicare or Medicaid, there are disparities in receiving ophthalmic services such as diabetic and glaucoma screenings [3, 4]. Some of the barriers contributing to socioeconomic and racial disparities in access to ophthalmic services include access to transportation, scheduling conflicts with work, out-of-pocket expenses for specialized services like glasses, and trust of healthcare providers [5–9].

As the American population continues to age, the need for ophthalmic services is increasing since aging is associated with a greater risk of blindness and vision impairment. Among this population, only approximately half received a dilated eye exam in the past 12 months. Another population at highest risk of ophthalmic complications are people with diabetes. Approximately one in three adults with diabetes consistently did not have a dilated eye exam in the past 12 months, even though this is recommended [9–11].

In order to serve the growing number of Americans who require ophthalmic services, there must be adequate training, including that of medical students. The amount of time that the curriculum of U.S. medical schools dedicate to ophthalmology education is decreasing which may be due in part to the absence of specific training guidelines by the Liaison Committee on Medical Education [12]. Between the second to fourth years of medical school, a longitudinal study showed that medical student skills in ophthalmic screening decreased due to the limited opportunities to reinforce these skills [13]. One solution is to enhance extracurricular exposure and experience in ophthalmology through student-run free clinics to provide access to ophthalmic care to those who lack access in the community under faculty supervision [12, 14, 15].

There are a limited number of examples of ophthalmology student-run free clinics, although the large majority of U.S. medical schools have student-run free clinics that provide healthcare to underserved populations as well medical student education [16, 17]. Student-run free clinics in ophthalmology can provide eye screening to uninsured and underinsured members of the community, including high-risk populations such as those without homes and those with diabetes, while providing clinical exposure and experience to medical students [14, 17–21]. This can also provide an opportunity for fourth year medical students to reinforce their ophthalmic skills by teaching their less experienced peers techniques such as the slit-lamp exam [15]. Medical students can also utilize these clinics to build valuable relationships with resident and attending ophthalmologists.

The Indiana University Student Outreach Clinic (IUSOC) Eye Clinic is a high-volume student-run free eye clinic that provides visual screening services to an underserved population in Indianapolis, IN, USA. In this retrospective study, we aimed to describe the patient demographic characteristics, review the visual screening services provided, illustrate a model for concurrent medical student education and involvement, and estimate the value of services provided to patients at our high-volume student-run eye clinic over seven years.

## Methods

This study was generated from the IUSOC Eye Clinic, a visual screening program that is run in partnership with the IUSOC, Indiana University School of Medicine (IUSM), and the Department of Ophthalmology at IUSM. Institutional review board (IRB) approval was obtained through Indiana University IRB. Patients were referred to the IUSOC Eye Clinic via the IUSOC medicine clinic or recruited via flyers. The IUSOC Eye Clinic was offered once a month for four hours during normal IUSOC clinic hours on a walk-in basis. Recruitment and eye exams were performed at the Neighborhood Fellowship Church in Indianapolis where the IUSOC medicine clinic and other services, such as legal and social work, are available once per week.

Eye screenings consisted of collecting demographic and medical history of the patients, followed by ocular screening performed by medical students, supervised by an IUSM ophthalmology resident and attending ophthalmologist. Eye examinations included distance visual acuity (VA) using a Snellen Chart, near visual acuity (NVA) using an automated vision screener, visual fields (VF) using a visual field analyzer, refraction using an auto-refractor, intraocular pressure (IOP) values collected by a Tono-Pen (Mentor Opthalmics, Norwell, MA, USA), fundus photography using a non-mydriatic fundus camera, and slit lamp examination. Screened conditions included refractive errors, cataracts, glaucoma, diabetic retinopathy and age-related macular degeneration. Results of the history and eye examination were documented in manual records and uploaded to the electronic medical record system, PracticeFusion (Practice Fusion Inc., San Francisco, CA, USA). Reading glasses were provided at no charge to those whose chief complaint was presbyopia. Patients who met the need for prescription eyewear were referred to two local LensCrafters retailers. LensCrafters is an international retailer of prescription eyewear with independent optometrists, and the IUSOC Eye Clinic has a partnership with two local retailers to provide free glasses to patients who present with a prescription from our clinic. Patients who met the need for further ophthalmologic care were referred to the local county hospital for further evaluation, diagnosis, and treatment.

We retrospectively reviewed the charts of 875 patients seen at the IUSOC Eye Clinic from October 2013 to February 2020. Data on demographics, insurance coverage, ocular history, examination, suspected diagnosis, referral status, and glasses provided was collected from patient charts. De-identified data was compiled and analyzed by the authors in a protected Excel (Microsoft Corporation, Seattle, WA, USA) spreadsheet following the Health Insurance Portability and Accountability Act of 1996 policies on electronic protected health information. For comparison, we retrospectively performed an analysis of the insurance coverage of patients seen at local university-affiliated facilities of Indiana University Health (IUH) ophthalmology clinics in Indianapolis from January 2018 through October 2021.

Relative value units (RVU) are an objective metric from the Center for Medicare & Medicaid Services to quantify the resources used to provide a medical service, including the time, intensity, and cost of care [22]. RVU are calculated from components related to the provider’s work and liability protection, the practice’s expenses, and are adjusted by geographic index to ensure alignment with the reimbursement system. RVU of the services provided at the IUSOC Eye Clinic were calculated from assigned work RVU with the specific Medicare administrative contractor (MAC) locality option 0810200 for Indiana on the 2021 Physician Fee Schedule from the Center for Medicare & Medicaid Services [22]. Similarly, the dollar value of services provided at the IUSOC Eye Clinic were calculated from the assigned non-facility price with the specific MAC locality option 0810200 for Indiana on the 2021 Physician Fee Schedule. The dollar value of services provided per screening was calculated by dividing the total dollar value of services provided by the total number of screenings.

The current procedural terminology (CPT) codes included for the assessment of services provided at our clinic are 92,002 (eye exam new patient, intermediate) for patient visits excluding those patients referred to county hospital ophthalmology service, 92,082 (visual field examination) for patients who received automated visual field testing, 92,250 (eye exam with photos) for patients who received fundus photography, and 99,202 (office or other outpatient visit for the evaluation and management of a new patient, 15–29 min) for patients referred to county hospital ophthalmology service. CPT code 92015 (determination of refractive state) is not covered by Medicare and, therefore, was not included in the calculations. CPT codes 92,002 and 92,004 both represent eye exams for new patients, with 92,002 being at an intermediate level and 92,004 at a comprehensive level. We chose the 92,002 code despite the supervising faculty ophthalmologist being qualified to provide comprehensive eye exams. Therefore, the RVU and dollar value calculations are underestimations of the total value of services provided at our clinic.

## Results

**Fig. 1:**
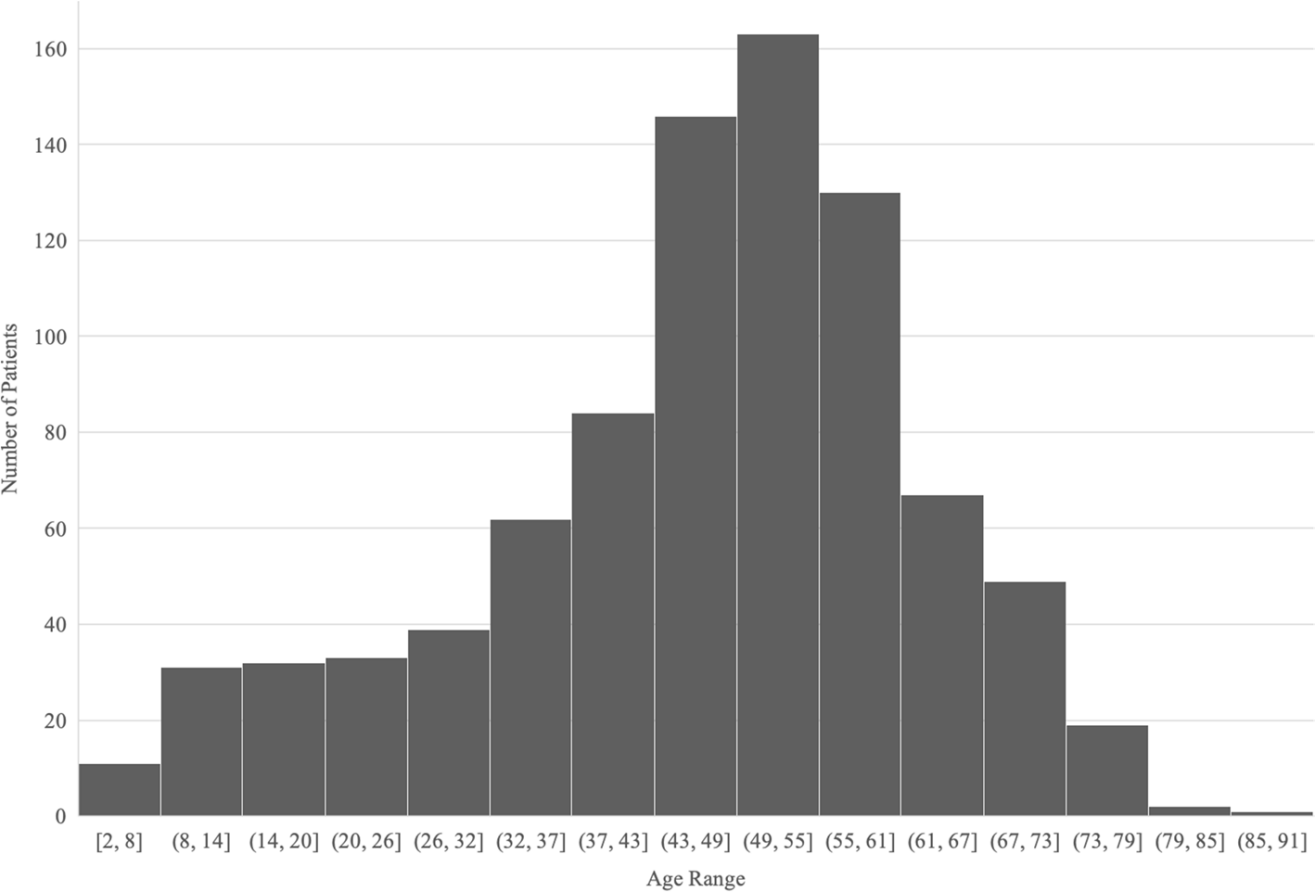
Age distribution of patients seen at the IUSOC Eye Clinic

875 patients were seen at the IUSOC Eye Clinic from October 2013 to February 2020. The demographic characteristics of these patients are presented in Table 1. The median was selected as the summary average statistic of the age of patients seen at the clinic due to the skewed distribution summarized in Fig. 1. Identified medical history, ocular history, and ophthalmic risk factors are also summarized in Table 1. The median was reported as the summary average statistic of years since diabetes diagnosis due to the non-parametric distribution of the data.

**Table 1:** Summary of patient characteristics

Median Age (IQR)	49 (18)						
Gender	Female: 447 (51.1%)		Male: 366 (41.8%)			Undocumented: 62 (7.1%)	
Ethnicity	African American: 279 (31.9%)	Caucasian: 190 (21.7%)	Hispanic: 188 (21.5%)	Other: 26 (3.0%)		Asian: 9 (1.0%)	Undocumented: 183 (20.9%)
Insurance	None: 343 (39.2%)	Medicaid: 105 (12.0%)	Medicare: 72 (8.2%)	Other: 42 (4.8%)	Healthy Indiana Plan: 31 (3.5%)	Private: 21 (2.4%)	Undocumented: 261 (29.8%)
Previous Visit	Yes: 68 (7.8%)		No: 218 (24.9%)			Undocumented: 589 (67.3%)	
Family History of Glaucoma	Yes: 143 (15.3%)		No: 691 (79.0%)			Undocumented: 50 (5.7%)	
Personal History of Glaucoma	Yes: 44 (5.0%)		No: 783 (89.4%)			Undocumented: 48 (5.5%)	
History of Eye Injury Or Surgery	Yes: 119 (13.6%)		No: 707 (80.8%)			Undocumented: 49 (5.6%)	
Recent Visual Change	Yes: 519 (59.3%)		No: 296 (33.8%)			Undocumented: 60 (6.9%)	
Recent Eye Pain	Yes: 150 (17.1%)		No: 628 (71.2%)			Undocumented: 97 (11.1%)	
Last Eye Exam Over 2 Years Ago	Yes: 453 (51.8%)		No: 377 (43.1%)			Undocumented: 45 (5.1%)	
Last Dilated Eye Exam Over 2 Years Ago	Yes: 478 (54.6%)		No: 330 (37.7%)			Undocumented: 67 (7.7%)	
Diabetes Diagnosis		Yes: 162 (18.5%)		No: 667 (76.2%)			Undocumented: 46 (5.3%)
	Median Years Since Diabetes Diagnosis (IQR)	6 (7)					
	Last Diabetic Eye Exam Over 1 Year Ago	Yes: 123 (75.9%)		No: 35 (21.6%)			Undocumented: 4 (2.5%)
Hypertension Diagnosis	Yes: 282 (32.2%)		No: 404 (46.2%)			Undocumented: 189 (21.6%)	

*Abbreviations*: *IQR* interquartile range

For insurance coverage of patients seen at the IUSOC Eye Clinic, 39.2% of the patients reported being uninsured, 12.0% reported having Medicaid insurance, 8.2% reported having Medicare insurance, 4.8% reported having other insurance, 3.5% reported having Healthy Indiana Plan (HIP) insurance, 2.4% reported having private insurance, and the insurance status of 29.8% patients was undocumented. Comparatively, for patients seen at IUH ophthalmology clinics, 1.3% of patients reported being uninsured, 16.4% reported having Medicaid insurance, 38.8% reported having Medicare insurance, 43.1% reported having private insurance, and 0.4% reported having other insurance.

**Fig. 2:**
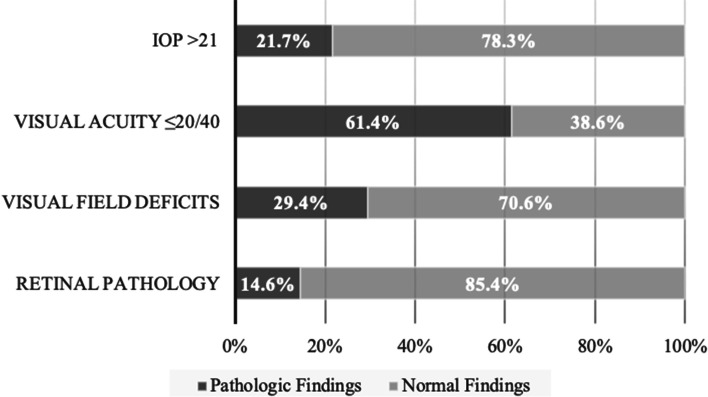
Summary of the screening findings of patients at IUSOC Eye Clinic

Results of the visual screening of patients seen at IUSOC Eye Clinic are presented in Table 2. The mean was reported as the summary average statistic for both IOP and cup-to-disc ratio due to the parametric distribution of the data. A summary of some of these visual screening results are also presented in Fig. 2.

**Table 2:** Summary of visual screening results

Mean IOP (±SD)	18.1 (±5.8)
IOP > 21	111 (21.7%)
Distance VA 20/40 or Worse	505 (61.4%)
Near VA 20/40 or Worse	323 (51.3%)
Visual Field Deficits	195 (29.4%)
Non-Mydriatic Fundus Photography	14.6% of all patients screened had evidence of retinal pathology 15.4% of diabetics screened had evidence of retinal pathology
Mean cup-to-disc ratio (±SD)	0.36 (±0.2)
Cup-to-disc ratio > 0.6	32 (8.3%)

*Abbreviations*: *IOP* intraocular pressure, *SD* standard deviation, *VA* visual acuity

**Fig. 3:**
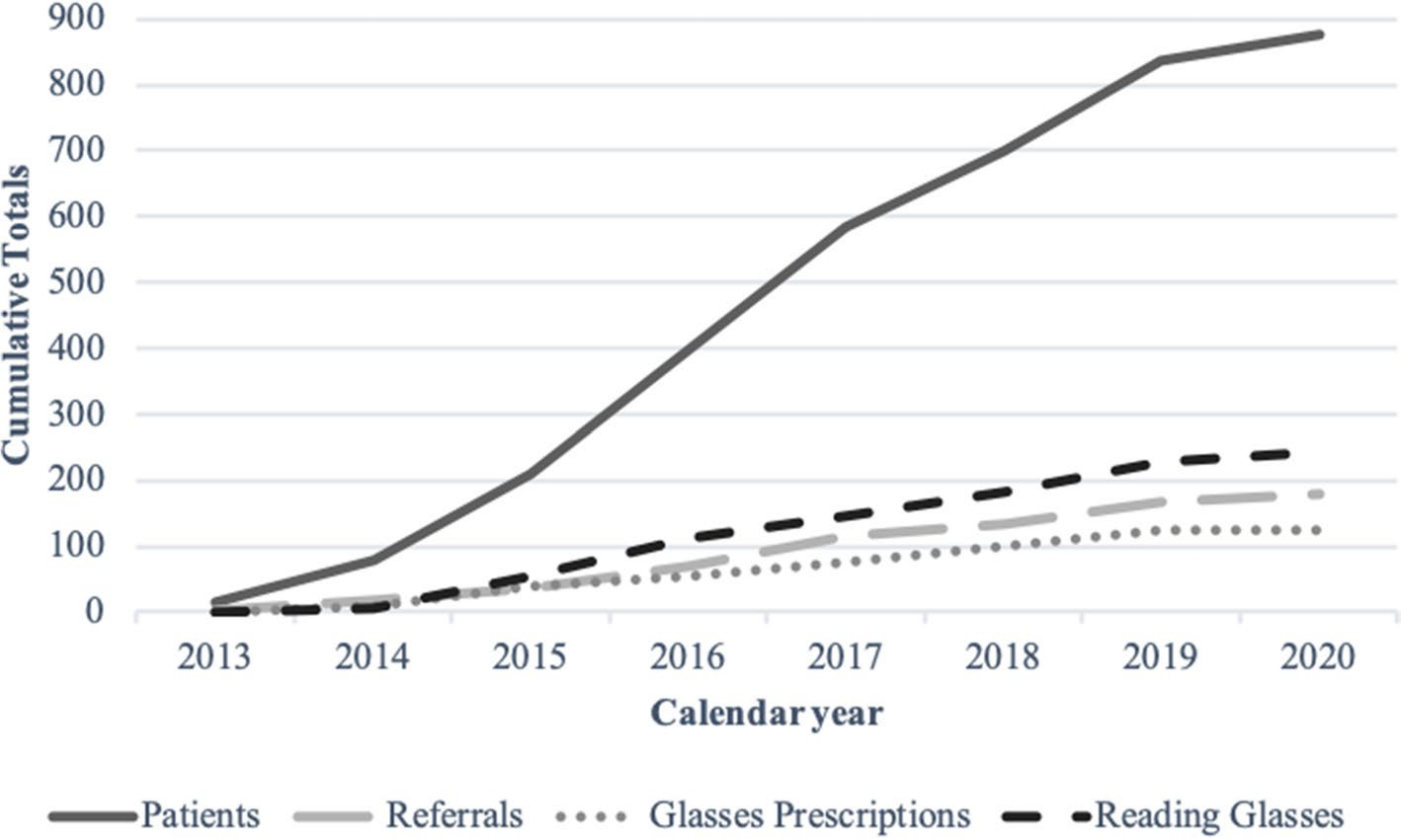
Cumulative totals of glasses provided and referrals for further care at the IUSOC Eye Clinic

The outcomes of patient visits, including reading glasses and glasses prescriptions provided, and referrals to local county hospital are summarized in Table 3 and Fig. 3. The estimated value of services provided at the IUSOC Eye Clinic from October 2013 to February 2020 was calculated to be 1271.3 RVU. The dollar value equivalent of services provided at our clinic was calculated to be $119,263.16, or $136.30 per screening.

**Table 3:** Summary of glasses provided and referrals for further care

Reading glasses provided	244 (27.9%)	
Glasses prescriptions provided	126 (14.4%)	
Referrals to ophthalmology service at county hospital	178 (20.3%)	
	Glaucoma	49 (5.6%)
	Decreased VA	39 (1.6%)
	Diabetic Retinopathy	14 (4.5%)
	Cataracts	11 (1.3%)
	AMD	5 (0.6%)
	Other Pathology	60 (20.3%)

*Abbreviations*: *VA* visual acuity, *AMD* age-related macular degeneration

## Discussion

In this study, we aimed to assess the community impact of our high-volume medical student-run free eye clinic over seven years. To the best of our knowledge, the IUSOC Eye Clinic is the largest student-run free eye clinic in the country based on the patient volume. Students, residents, and faculty were able to provide care to 875 patients over the seven-year period. 244 patients received free reading glasses, 126 patients received free glasses prescriptions, and 178 patients were referred for more advanced care for ophthalmic pathology, including glaucoma, diabetic retinopathy, cataracts, and age-related macular degeneration. In summary, the clinic has provided critical eye care to the patient population on the Near East Side of Indianapolis.

A distinguishing feature of our clinic is the large variety of patients that present to the clinic, including patients with an expansive age distribution, differing socioeconomic and medical backgrounds, and a wide range and severity of symptoms and pathology. The clinic’s free vision screening services were especially valuable to uninsured or underinsured patients without readily accessible ophthalmic care. In particular, nearly 40% of patients were uninsured, with the true figure likely being significantly higher given that an additional 30% had undocumented insurance status. Contrarily, only 1.3% of patients seen at the local IUH ophthalmology clinics were uninsured. The proportion of uninsured patients seen at our outreach clinic is also more than triple that of the total proportion of uninsured nonelderly individuals in the state of Indiana, reported to be 10.3% in 2019 [23]. However, it appears to compare similarly to other community eye clinics that have reported on the insurance status of the patient population they serve [24, 25]. The large proportion of uninsured patients seen at the IUSOC Eye Clinic is especially notable considering the lack of insurance has been well-reported to result in decreased access to and utilization of eye care services, thereby increasing the risk of irreversible visual impairment [10, 26, 27].

Students were also provided the opportunity to serve a diverse patient population and receive exposure to a broad range of pathology. The median patient age was 49 years old, with a wide range of age 2 to 91. Patients of many different races and ethnicities received care at our clinic, exposing students to unique cultural and genetic risk factors. The screening program was able to detect a significant amount of pathologic ophthalmic findings that may otherwise have went undetected and untreated. Patients presented with a wide range of ophthalmic disease, from refractive error and presbyopia to cataracts, glaucoma, diabetic retinopathy, age-related macular degeneration and other pathology, such as trauma, strabismus, and pterygia. Over seven years, the cumulative value of services provided at our clinic was estimated to be 1271 RVU. This is equivalent to an estimated healthcare cost savings of nearly $120,000, or $136 per screening. Thus, our clinic has played a critical role in providing free, basic eye care to a diverse, inner-city community with prevalent ocular pathology.

While the IUSOC Eye Clinic holds community service at the core of its mission, equally important is the clinic’s educational mission. Ophthalmology as a specialty is underemphasized in the general medical school curriculum, making it difficult for interested medical students to gain exposure to the field. Our student-run clinic addresses this gap by enabling students to learn firsthand about ophthalmology. The IUSOC Eye Clinic is operated by IUSM medical students from the Ophthalmology Student Interest Group (OSIG) under the supervision of ophthalmology attending physicians and upper-level residents. One attending ophthalmologist and one resident ophthalmologist supervise approximately five to ten medical students at each clinic, offering the opportunity for involvement to upwards of 100 students every year. Medical students are in charge of recruiting student and physician volunteers, applying for grants in support of the clinic, maintaining the inventory of delicate eye exam equipment, scheduling and seeing patients in clinic, referring patients in need of advanced ophthalmic care to the county hospital, and coordinating care with other IUSOC services, including Medicine, Social Work, and Spanish translation. Furthermore, students may contribute to quality improvement research projects to enhance the clinic’s workflow. Thus, the clinic provides a much-needed opportunity for medical students to engage in community service, organizational leadership, and networking – all while gaining valuable medical experience in their field of interest.

This study of our community eye clinic has a number of limitations. The retrospective study revealed significant incomplete documentation of patient histories and encounters, affecting the data collection and analysis by underreporting. Furthermore, the accuracy of the data was likely limited by the reliance on self-reported history from patients. Documentation of the self-reported history, screening findings, and examination results also likely differed among the medical students performing the encounters. Finally, while our clinic provides an important opportunity to meet the educational needs of students and the clinical needs of patients, this study does not include objective assessments of students’ educational benefits or patients’ satisfaction with the clinic. Future research studies could incorporate student surveys about what they learned and their ratings of the educational value of the clinic, a pre- and post-assessment to evaluate students’ knowledge and skills, and patient surveys about their experience.

Additionally, the current operation of the IUSOC Eye Clinic has multiple limitations. First, the clinic is financed by the IUSM OSIG medical student organization with a modest annual budget of several thousand dollars. The clinic’s operations are funded solely by grants and occasional donations of eye exam equipment and reading glasses. Thus, the current budget limits the scalability of our clinic and our ability to purchase newer, costly equipment. In addition, the eye clinic has limited hours of operation. The clinic is open for four hours at one location on one Saturday per month. Although approximately 20 patients on average are seen per clinic date, our monthly clinic may not be a feasible option for patients requiring more immediate eye care. Expanding operation to several dates per month would be challenging, given the need for available student and physician volunteers and the need to coordinate shared clinic space with other IUSOC services. Furthermore, limited patient transportation and single community location prevents the clinic from serving more people. Given the lower socioeconomic status of the population served, many patients arrive to clinic by foot. Thus, patient turnout is generally limited to nearby residents on the east side of Indianapolis and may fluctuate with inclement weather. Finally, our clinic provides basic vision screening and diagnostic services but is not equipped to treat advanced ocular pathology. Thus, patients requiring follow-up eye care must be referred to the ophthalmology clinic at the local county hospital. Future direction for the clinic includes improving the referral process, expanding the clinic services to other areas of Indianapolis or other cities in Indiana, exploring a mobile clinic format, and establishing virtual visit options in the era of COVID-19, as well as to address patient transportation barriers.

## Conclusions

In conclusion, we hope that our student-run free eye clinic will serve as a model for other free clinics nationwide that provide ophthalmic or medical care to their local communities. We have demonstrated that a student-run free eye clinic can provide critical vision screening services to uninsured and underinsured patients, while simultaneously providing an educational experience for medical students pursuing careers in ophthalmology. In providing basic eye care to nearly 900 patients since 2013, the IUSOC Eye Clinic fills an important role in advancing ocular health and preventing irreversible blindness in the underserved Indianapolis community.

## Data Availability

Not applicable.

## Abbreviations

IUSOC: Indiana University Student Outreach Clinic
IUSM: Indiana University School of Medicine
IRB: Institutional review board
VA: Visual acuity
NVA: Near visual acuity
VF: Visual fields
IOP: Intraocular pressure
IUH: Indiana University Health
RVU: Relative value units
MAC: Medicare administrative contractor
CPT: Current procedural terminology
HIP: Healthy Indiana Plan
OSIG: Ophthalmology Student Interest Group
